# Genome-wide Identification of TCP Family Transcription Factors from *Populus euphratica* and Their Involvement in Leaf Shape Regulation

**DOI:** 10.1038/srep32795

**Published:** 2016-09-08

**Authors:** Xiaodong Ma, Jianchao Ma, Di Fan, Chaofeng Li, Yuanzhong Jiang, Keming Luo

**Affiliations:** 1 Key Laboratory of Adaptation and Evolution of Plateau Biota, Northwest Institute of Plateau Biology, Chinese Academy of Sciences, Xining, 810008, China; 2 School of Chemistry and Chemical Engineering, Qinghai University for Nationalities, Xining, 810007, China; 3 University of the Chinese Academy of Sciences, Beijing, 100049 China; 4 State Key Laboratory of Grassland Agro-Ecosystem, School of Life Science, Lanzhou University, Lanzhou, China; 5 School of Life Science, Southwest University, Chongqing, 400715 China

## Abstract

Higher plants have been shown to experience a juvenile vegetative phase, an adult vegetative phase, and a reproductive phase during its postembryonic development and distinct lateral organ morphologies have been observed at the different development stages. *Populus euphratica*, commonly known as a desert poplar, has developed heteromorphic leaves during its development. The TCP family genes encode a group of plant-specific transcription factors involved in several aspects of plant development. In particular, TCPs have been shown to influence leaf size and shape in many herbaceous plants. However, whether these functions are conserved in woody plants remains unknown. In the present study, we carried out genome-wide identification of TCP genes in *P. euphratica* and *P. trichocarpa*, and 33 and 36 genes encoding putative TCP proteins were found, respectively. Phylogenetic analysis of the poplar TCPs together with Arabidopsis TCPs indicated a biased expansion of the TCP gene family *via* segmental duplications. In addition, our results have also shown a correlation between different expression patterns of several *P. euphratica* TCP genes and leaf shape variations, indicating their involvement in the regulation of leaf shape development.

Higher plants usually experience three postembryonic developmental stages, i.e. a juvenile vegetative phase, an adult vegetative phase, and a reproductive phase^1^,^2^. These phases are defined by the morphology of lateral organs^3^, which is marked by a number of either continuous or discrete features. As a major lateral organ, leaf shows both continuous and discrete morphological changes in phase transition: leaf size shows a continuous variation, while several distinct shapes are seen in different plant developmental phases^4^. Phase transition has been shown to be regulated by both environmental factors such as photoperiod^5^,^6^ and intrinsic regulators including plant hormones such as gibberellins^6^, *trans*-acting small interfering RNAs (tasiRNAs)^7^,^8^, and most importantly, microRNAs^9^. MiR156, a conserved microRNA among both herbaceous and woody plants which shows response to sugar concentration^10^, as well as its downstream regulatory module components such as SQUAMOSA promoter-binding protein-like (SPL) genes and MiR172, has been shown as responsible for development phase transition, and thus controlling leaf morphology changes in Arabidopsis, maize, and several woody species including *Eucalyptus globulus*, and *Populus* x *Canadensis*^11^,^12^,^13^,^14^,^15^. However, more details of the downstream regulation cascade for controlling leaf shape remain to be elucidated.

The process of leaf development is composed of primordia initiation, establishment of polarity in three axes, lamina expansion, and formation of leaf margin^16^,^17^,^18^, and involves coordinated regulation among transcription factors, small RNAs and hormones^19^. Genetic approaches have demonstrated several key genes involved in leaf development. For example, suppression of the expression of Class I KNOTTED1-LIKE HOMEOBOX (KNOX) genes has been shown as critical for leaf primordia initiation^20^,^21^. Class III HD-ZIP, KANADI and YABBY gene families are involved in the establishment of dorsoventral polarity^22^,^23^,^24^,^25^,^26^,^27^. ANGUSTIFOLIA (AN) and ROTUNDIFOLIA3 (ROT3), and ANGUSTIFOLIA3 (AN3) and ROTUNDIFOLIA4 (ROT4) affected lamina expansion by regulating the shape and number of cells in the lamina, respectively^17^. In addition, PIN and CUC genes play crucial role in leaf margin patterning by controlling auxin-maxima formation^28^,^29^.

The TCP transcription factor family, named after its initial members teosinte branched1 (*tb1*) from maize (*Zea mays*)^30^, CYCLOIDEA (CYC) from snapdragon (*Antirrhinum majus*)^31^, and the PROLIFERATING CELL FACTORS 1 and 2 (PCF1 and PCF2) from rice (*Oryza sativa*)^32^, was first described in 1999, as a small group of plant specific genes encoding proteins sharing the so-called TCP domain, which is a 59-amino acid non-canonical basic helix-loop-helix (bHLH) motif that allows DNA binding, protein-protein interactions and nuclear localization^32^,^33^,^34^. TCPs have been identified in various plants including Arabidopsis^35^, rice^36^,^37^,^38^, tomato^39^ and poplar^34^,^37^. These genes have been categorized by phylogenetic analysis into two classes based mainly on differences within the TCP domain: class I (known as PCF class or TCP-P class) and class II (known as TCP-C class)^33^,^37^,^40^. Class I is formed by a group of relatively closely related proteins exemplified by rice PCF1 and PCF2, whereas class II is further subdivided into two clades according to differences within the TCP domain: the CIN clade exemplified by CINCINNATA (CIN) of *Antirrhinum*^41^ and the CYC/TB1 clade (or ECE clade) including CYC and tb1^42^.

Many members of the TCP family from both Class I and Class II have been demonstrated to participate in leaf development control in *Antirrhinum*^41^, Arabidopsis^43^,^44^,^45^,^46^,^47^,^48^,^49^, and tomato^50^. They have been shown to physically interact with several proteins involved in leaf development regulation, including ASYMMETRIC LEAVES1 (AS1), AS2 and NGATHA (NGA)^46^,^51^,^52^. In particular, SPL9, a target of MiR156, has been reported to interact with TCP4 and this complex promoted CUC-controlled acquisition of leaf complexity in Arabidopsis^53^, indicating that TCPs may play important roles in the regulatory cascade of leaf shape control centered by the MiR156 module. However, it remains unknown whether TCPs are also involved in leaf morphology regulation in woody plants.

With its genome well sequenced and annotated^54^, *P. trichocarpa* (black cottonwood) has been widely employed as a model tree for genomic and genetic studies. *P. trichocarpa* possesses almost identical leaf shapes during development phase transitions. In contrast, the desert poplar *P. euphratica* employs linear-laceolate, lanceolate, oval and wide-oral leaves sequentially along the process of development with a dramatic increase in leaf area, thickness, dry weight, as well as specific leaf area^55^,^56^,^57^. Recently, the genome sequence of *P. euphratica* has also been sequenced and assembled^58^,^59^, making it a suitable model for studying the roles of TCP transcription factors in leaf morphology establishment at genome-wide scale. Therefore, in this study, we firstly carried out genome-wide identification of TCP transcription factors in *P. trichocarpa* and *P. euphratica*, and then we conducted expression analysis of TCP genes in various leaf types of *P. euphratica* to establish a correlation between their expression and various leaf morphologies.

## Results

### Identification of TCP genes in *P. trichocarpa* and *P. euphratica*

We first performed genome-wide search of putative TCP genes in both *P. trichocarpa* and*. P. euphratica*. At least 60 putative TCP proteins were identified in the *P. trichocarpa* genome, all of which were annotated as TCP family protein in the Plant Transcription Factor Database 3.0^60^, confirming the reliability of our initial search. Among them, 23 were identified as redundant sequences resulting from alternative splicing and thus discarded. Multiple-alignment was carried out for the remaining protein sequences together with the Arabidposis TCPs. Manual inspection on the alignment revealed one peptide sequence without a TCP domain which was thus discarded. The 36 putative TCPs genes were named *PtrTCP1* to *PtrTCP36* in the order of their access number in the Phytozome database^61^ (Supplementary Table S1). Similar HMMER search and manual inspection were conducted against the *P. euphratica* genome and 33 non-redundant putative genes were identified and named as *PeuTCP1* to *PeuTCP33* according to their accession number (Supplementary Table S2). These poplar TCPs have a peptide length ranging from 41 to 623 amino acids, a molecular weight between 4787.43 and 68064.55, and an isoelectric point (pI) value between 5.84 and 9.58 (Supplementary Fig. S1, Supplementary Tables S1 and S2). The molecular weight and pI values of these poplar TCPs showed similar distribution as those of Arabidopsis TCPs (Supplementary Fig. S1).

### Phylogenetic analysis of TCP proteins

**Figure 1:**
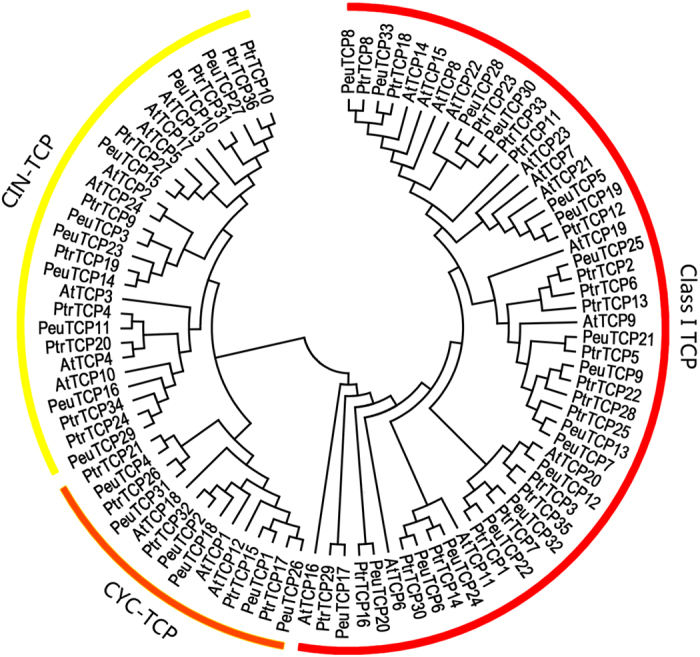
Phylogenetic tree of *Arabidopsis* and *Populus* TCPs. The phylogenetic tree was built based on multiple alignment of the TCP domain in the TCP proteins using the Neighbor-Joining method with 1000 bootstrap replicates.

In order to elucidate phylogenetic relations among the poplar TCPs, a maximum likelihood (ML) phylogenetic tree was built based on multiple-alignment of the TCP domain sequences of the poplar TCPs and their Arabidopsis homologues. As shown in Fig. 1, the 93 TCPs were classified into two classes, Class I (red) and Class II, where Class II was further divided into two clades, CYC (orange) and CIN (yellow). All Arabidopsis TCPs fell in the same Class or clade as previously reported^34^, confirming the reliability of our phylogenetic tree. Similar phylogenetic trees were obtained by the minimal evolution, maximal parsimony, neighbour joining methods with minor differences at some branches (Supplementary Figs S2–S4). According to the phylogenetic tree, both poplar species possessed an expanded TCP family with approximate 1.5-fold size compared with Arabidopsis. Interestingly, the expansion in both poplar species was biased, which occurred mainly in Class I and the CYC clade, while the CIN clade remained largely the same size as in Arabidopsis (Table 1).

### Chromosomal location analysis of *PtrTCP* genes

**Figure 2:**
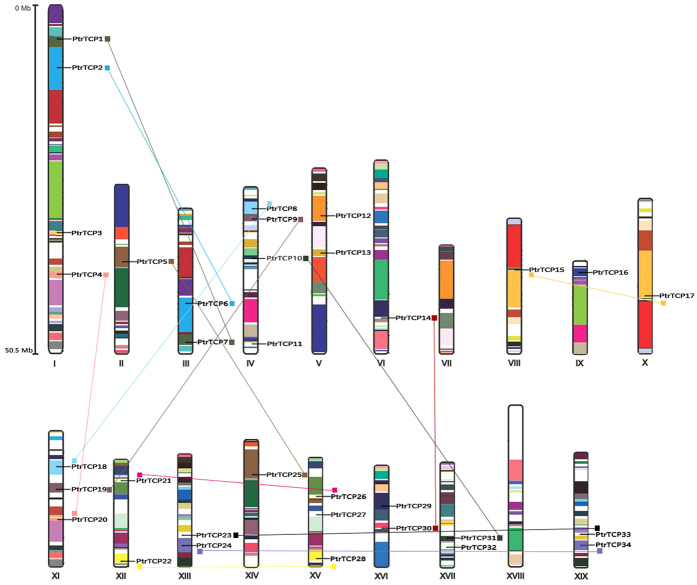
Physical locations of TCP genes on *P. trichocarpa* chromosomes. The TCP genes are located according to the JGI *P. trichocarpa* genome V. 3.0 annotations, and possible gene duplication events are indicated by colored lines.

Among the 36 *P. trichocarpa* TCPs, 34 members were located at the 19 chromosomes assembled in the *P. trichocarpa* genome v 3.0, and the other two were located at two unmapped scaffolds, *viz.* Scaffold 41 and Scaffold 457, respectively. TCP genes are distributed on 17 out of the 19 *P. trichocarpa* chromosomes in an uneven manner, with the number of TCP genes per chromosome ranging from 0 to 4. Chromosomes 1 and 4 contain four genes, while no TCP gene is found on Chromosomes 7 and 18 (Fig. 2). Additionally, as shown in Fig. 2, 13 pairs of duplicated genes were identified, accounting for about 72% of the *P. trichocarpa* TCP family, complying with the ~1.5-fold expansion of the poplar genome as compared with Arabidopsis^54^. In fact, as the two genes located to unmapped scaffolds also show high identity to other genes, there could be even more duplication events. Further, all the paralogous gene pairs we identified located on different chromosomes, suggesting that they result from segment duplications rather than tandem duplications.

### Genomic structure of the TCP genes

**Figure 3:**
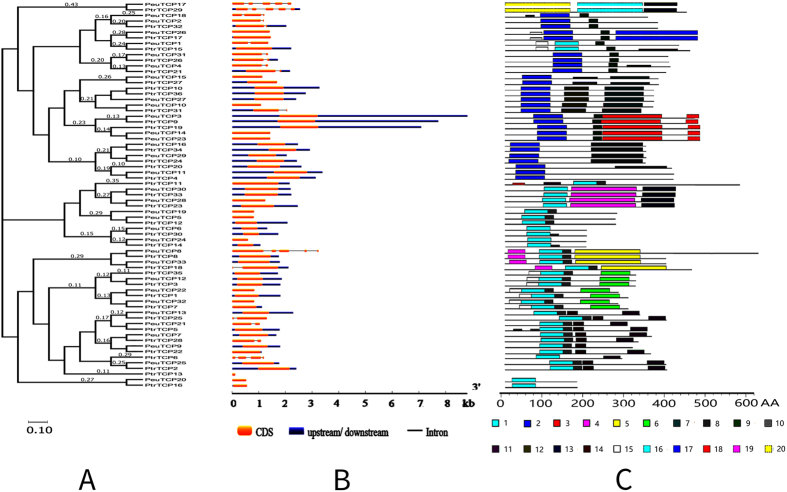
Genomic structure and motif composition of poplar TCPs. (**A**) Phylogenetic tree of *P. trichocarpa* and *P. euphratica* TCP proteins. (**B**) Genomic structure of poplar TCP genes. Exons, introns, and UTRs are indicated with yellow boxes, blue boxes and black lines, respectively. (**C**) Motif composition of poplar TCP proteins. Conserved motifs in the poplar TCP proteins are indicated by colored boxes.

As shown in Fig. 3B, most (53 out of 69) of the poplar TCP genes contain only one exon and 11 members contain one intron and two exons. Only *PeuTCP18* compromises two introns and three exons and *PtrTCP6* possesses three introns and four exons, whereas three genes (*PeuTCP17*, *PtrTCP29* and *PeuTCP8*) consist of four introns and five exons. Moreover, similar exon/intron structures were found in poplar genes within the same phylogenetic subfamily, further confirming the reliability of our phylogenetic analysis.

### Prediction of conserved motifs in TCP proteins

Conserved motifs in the poplar TCP proteins were analyzed with the MEME program^62^ and shown in Fig. 3C. The MEME analysis discovered 20 putative motifs in total, namely motif 1 to motif 20 (Supplementary Fig. S5). Motif 1 and Motif 2 were both identified as the conserved TCP domain, while no matches were found for the other motifs. Either motif 1 or motif 2 is present in every poplar TCP protein we have identified, providing further support for the reliability of our identification. Additionally, although the functions of the rest 18 motifs are unknown, similar motif composition was observed in TCP proteins of the same subfamily, while significant differences were observed between different subfamilies, indicating possible intra-subfamily functional redundancy and inter-subfamily function divergence.

### Global expression profiling in *P. trichocarpa*

**Figure 4:**
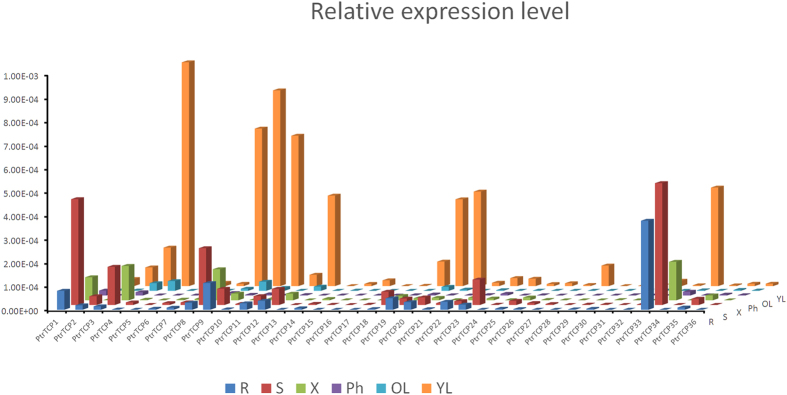
Transcriptional profiling of *P. trichocarpa* TCP genes in root, stem, xylem, phloem, old leaf and young leaf. Each bar represents three biological replicates.

Relative transcript abundance of the 36 *PtrTCP*s in six *P. trichocarpa* tissues are shown in Fig. 4. Generally, 18 out of the 36 genes showed high expression levels in the young leaf, four genes (*PtrTCP1*, *PtrTCP23*, *PtrTCP33* and *PtrTCP35*) with high expression levels in the stem, and one gene (*PtrTCP33*) with high level of expression in the root. Moreover, poplar TCP genes with close phylogenetic relationship showed both similar and divergent expression patterns. For example, the paralogous pair *PtrTCP9* and *PtrTCP19* were both expressed highly in the young leaf, at a moderate level in the root, and at a low level in the stem, and the expression level of *PtrTCP9* was higher than that of *PtrTCP19* in each tissue we tested. In contrast, another paralogous pair, *PtrTCP1* and *PtrTCP7*, divergent expression patterns were found. *PtrTCP7* was almost undetectable in all tissues we examined, while *PtrTCP1* was expressed highly in the stem, and moderately in the root and xylem.

### Correlation of *TCP* expression levels and leaf morphogenesis in *P. euphratica*

**Figure 5:**
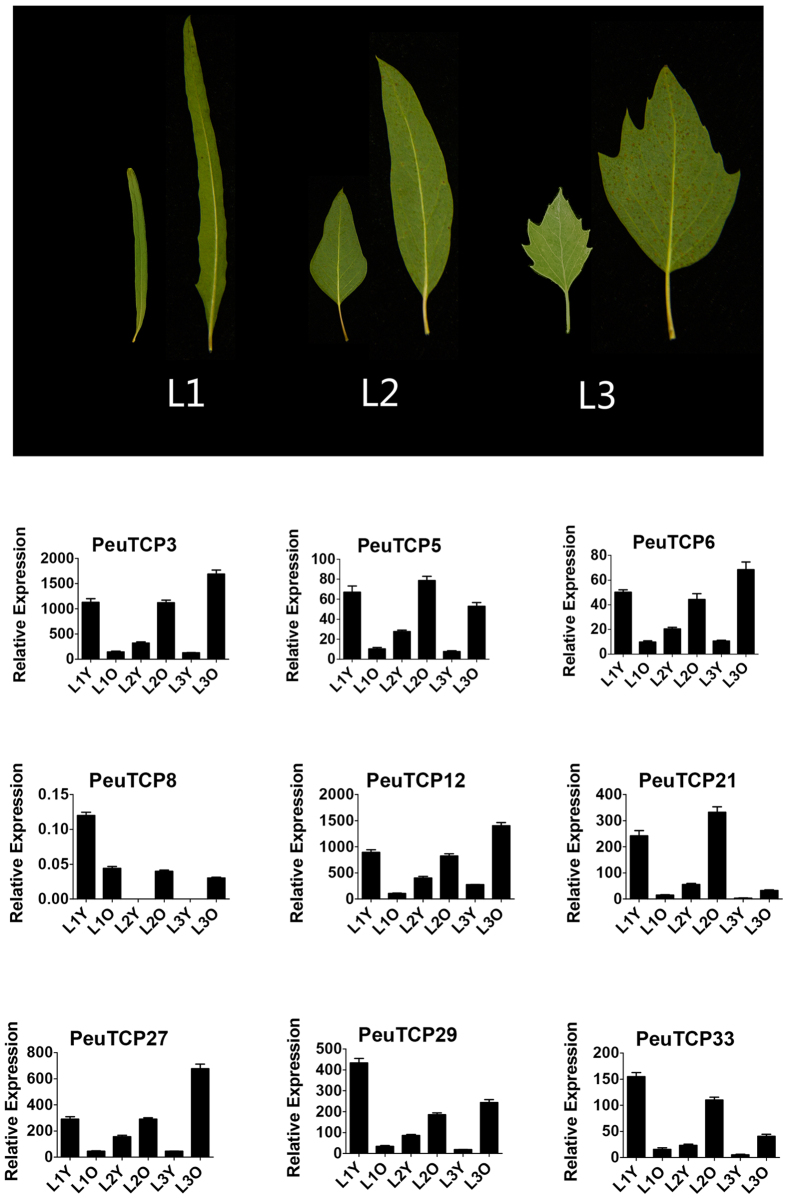
Expression pattern of selected TCP genes in different *P. euphratica* leaves. Each bar represents three biological replicates and error bar represents S.D.

In *P. euphratica*, linear-laceolate leaves are found in juvenile (first year) shoots, and lanceolate and oval leaves are sequentially seen later in adult phase shoots (Fig. 5, top panel). Previous studies have shown that *A. thaliana* TCPs from Class I and the CIN clade, but not from the CYC clade, i.e. *AtTCP2*, *AtTCP3*, *AtTCP4*, *AtTCP10*, *AtTCP24*, *AtTCP5*, *AtTCP13*, *AtTCP7*, *AtTCP21*, *AtTCP23*, *AtTCP9*, *AtTCP20*, *AtTCP11*, *AtTCP14* and *AtTCP15*^43^,^44^,^45^,^46^,^47^,^48^,^49^ are involved in leaf morphology regulation. Based on our phylogenetic analysis as well as transcriptome sequencing data previously reported^58^, we selected 9 representative *TCP* genes that are both closely related to the Arabidopsis TCPs motioned above and highly expressed according to the transcriptome analysis, i.e. *PeuTCP3* (homologous to *AtTCP2* and *AtTCP24*), *PeTCP5* (homologous to *AtTCP7*, *AtTCP21* and *AtTCP23*), *PeuTCP6* (homologous to *AtTCP11*), *PeuTCP8* (homologous to *AtTCP14* and *AtTCP15*), *PeuTCP12* (homologous to *AtTCP2*0), *PeuTCP21* (homologous to *AtTCP9*), *PeuTCP27* (homologous to *AtTCP5* and *AtTCP13*), *PeuTCP29* (homologous to *AtTCP3*, *AtTCP4* and *AtTCP10*) and *PeuTCP33* (homologous to *AtTCP14* and *AtTCP15*) and performed qRT-PCR in *P. euphratica* leaves with different shape to examine expression patterns of *TCP* genes during leaf development. In general, *TCP* members of both Class I and Class II showed a decrease of expression level in young leaves along the developmental process (Fig. 5, bottom panel). Interestingly, two different expression patterns were observed in linear-laceolate leaves and the other two-type leaves. In the linear-laceolate leaves (L1), the expression levels for all the *TCPs* were higher in young leaves than adult ones, while lower expression levels were observed in young lanceolate and oval leaves than corresponding adult leaves (L2 and L3).

To determine whether such expression patterns are specifically correlated with the leaves with different shape, we conducted similar qRT-PCR test for *TCP* genes in *P. tomentosa* leaves from juvenile and adult shoots, as this poplar species employs a single leaf shape during its whole life span.The expression patterns of *PtoTCP* genes were different from their homologues in *P. euphratica*: higher expression levels were observed in young leaves than adult leaves from both juvenile (one year) and adult phase (multiple year) shoots (Supplementary Fig. S6). In addition, as SPLs have been shown as important developmental markers in both Arabidopsis^13^ and hybrid poplar^14^, we also conducted qRT-PCR analysis for *P. euphratica* SPL genes homologous to *AtSPL3* and *AtSPL9*, i.e. *PeuSPL6* and *PeuSPL23*, and *PeuSPL8, PeuSPL17* and *PeuSPL27*, respectively. These SPL genes showed similar expression patterns as the TCP genes in *P. euphratica*. (Supplementary Fig. S7).

## Discussion

TCP family transcription factors are plant specific transcription factor that play various roles in multiple aspects of plant growth and development. In the present study, we identified and characterized genes encoding TCP proteins in *P. euphratica* and *P. trichocarpa*. The TCP family was expanded by ~1.5 fold in both poplar species compared with Arabidopsis and the expansion was due to segmental duplications rather than tandem duplications. Further, as indicated by phylogenetic analysis, such expansion was carried out in an uneven manner that Class I and the CYC clade TCPs were expanded in poplar, while the number of CIN clade TCPs remained almost the same as in Arabidopsis. Moreover, while 23 out of 33 PeuTCPs were clustered closely to their *P. trichocarpa* orthologues, the others suggested both putative gene duplication and gene loss events in either species. For example, PtrTCP3 and PtrTCP35, PtrTCP10 and PtrTCP36, PeuTCP7 and PeuTCP13 were shown as closest homologs to each other, indicating possible gene duplications in the respective species. Meanwhile, the orthologues of PtrTCP5, PtrTCP6, PtrTCP13, PeuTCP18 and PeuTCP18 were missing, suggesting the occurrence of gene loss events in the divergence of the two poplar species.

In Arabidopsis, several TCP transcription factors from both Class I and Class II have been shown to participate in the regulation of leaf shaping. For example, dominant negative repression of AtTCP11 led to curled rosette leaves^47^, and AtTCP14 and AtTCP15 have been shown to influence leaf cell size and number in a redundant manner^48^. In addition, partial redundancy and functional divergence in the regulation of leaf shape was found among Arabidopsis TCPs with close phylogenetic relationship. For instance, members of the CIN Clade, i.e. AtTCP2, AtTCP3, AtTCP4, AtTCP10 and AtTCP24 are shown to influence leaf shape in a complementary manner^4^,^63^,^64^,^65^. Therefore, *P. euphratica* TCPs with close phylogenetic relationship to these AtTCPs could also play a role in the regulation of leaf shape, and functional divergence could be expected.

Moreover, we discovered a correlation between the expression pattern of *TCP* genes and leaf shape regulation in *P. euphratica*, and such altered changes were also observed for *SPL* genes, whose homologues had been established as developmental markers^13^,^14^, indicating their possible involvement in the developmental transition. Changes in the expression pattern of *TCP* genes in different *P. euphratica* leaves suggests the involvement of *P. euphratica TCP* genes from both Class I and Class II in leaf shape regulation. However, their regulatory roles could be divergent. For example, it has been shown that Arabidopsis Class II TCP *AtTCP4* regulates the serration at the leaf margin by influencing auxin distribution *via* the miR164-CUC pathway^63^. *PeuTCP29*, the closest *P. euphratica* homologue of *AtTCP4*, could play similar role in leaf development process. We speculate that, at the juvenile stage, high level of *PeuTCP29* in the young leaf represses serration at the leaf margin, and when the leaf grows older, the expression of *PeuTCP29* is reduced, resulting in formation of mild serrations. While at the adult phase, the expression of *PeuTCP29* is repressed in the young leaf, leading to formation of deep serrations, and in old leaf, although the repression is somehow removed, the serrations have been established. Other Class II *P. euphratica TCP* genes (*PeuTCP3* and *PeuTCP27*) could play similar role as *PeuTCP29*. On the contrary, Class I *TCP* genes have been shown to influence cell growth and proliferation, and their altered expression in the leaf could result in changes in cell number and size^48^,^66^, or the disruption of the balance between the growth rate in inner regions of the leaf lamina and that at the leaf margins, leading to curled leaf shape^47^. Therefore, the inverted expression pattern of Class I *P. euphratica TCP* genes (*PeuTCP5*, *PeuTCP6*, *PeuTCP8*, *PeuTCP12*, *PeuTCP27* and *PeuTCP33*) could be correlated to the altered length-width ratio of *P. euphratica* leaves. However, the detail functions of these genes in *P. euphratica* still need to further determine in the future.

## Materials and Methods

### Sequence retrieval and TCP gene identification

The Hidden Markov Model (HMM) profile of the TCP domain (PF03634) was obtained from the pfam database^67^ and putative TCP protein sequences were identified by HMMER^68^ search against the *P. euphratica* genome^58^ and the publicly available *P. trichocarpa* genome v 3.0^54^ in the Phytozome database^61^ with the HMM profile. In cases of multiple putative proteins from the same gene locus, the longest variant was kept for further analysis.

### Phylogenetic analysis

Amino acid sequences (full-length sequences or the TCP domain sequences) of the putative TCP proteins were used for multiple alignment and phylogenetic analysis with the program MEGA version 6^69^ with default settings.

### Analysis of protein features

Molecular weights and theoretical isoelectric point (pI) values of the TCP proteins were calculated with the ProtParam program at the ExPAsy bioinformatics resource portal^70^. The peptide sequences of the poplar TCPs were submitted to the Multiple Em for Motif Elicitation (MEME) server^62^ and motif discovery was done with the following settings: motif discovery mode: normal mode; site distribution: any number of repetitions; number of motifs: 20; background motif: 0-order model of sequences; motif width: minimum: 6, maximum: 200. The motifs identified by the MEME analysis were searched in the PROSITE database^71^.

### Analysis of genomic organization

Chromosome size and chromosomal location information for the *PtrTCP* genes was obtained from the Phytozome database^61^, and the genes were mapped to each chromosome proportionally.

Genomic and CDS sequences of the poplar TCPs were submitted to the Gene Structure Display Server (GSDS) 2.0^72^ and the output gene structures were arranged according to a phylogenetic tree of the poplar TCPs. Paralogous gene pairs were identified according to genomic sequence identity, and the following criteria were employed: the shorter sequences should cover at least 70% of the longer sequence and the identity of aligned regions should be no less than 70%^73^.

### RNA isolation and quantitative RT-PCR

*P. trichocarpa* materials were collected from wild-type plants grown in the greenhouse of School of Life Science, Southwest University, Chongqing with three biological replicates. *P. euphratica* leaves were collected from 6-year old trees grown in the common garden, Lanzhou University, different leaves from the same tree were used for comparison and leaves series from three different trees were taken as biological replicates. Total RNA was isolated from various tissues of *P. trichocarpa* and *P. euphratica* with a BioFlux Biospin Plant Total RNA Extraction Kit (Bioer Technology) following the manufacturer’s instructions and reverse-transcription was performed with a PrimeScript RT reagent Kit with gDNA Eraser (47A) (Takara, Dalian). Quantitative real-time RT-PCR (qRT-PCR) was carried out on a Takara Thermal Cycler Dice real time system (Takara, Japan) with a GoTaq qPCR Master Mix (Promega). Primers used in in this work are listed in Supplementary Table S3.

## Additional Information

**How to cite this article**: Ma, X. *et al*. Genome-wide Identification of TCP Family Transcription Factors from *Populus euphratica* and Their Involvement in Leaf Shape Regulation. *Sci. Rep.* **6**, 32795; doi: 10.1038/srep32795 (2016).

## Supplementary Material

Supplementary Information

## Figures and Tables

**Table 1:** TCP genes in *Arabidopsis* and the two *Populus* species.

Species	Class I TCPs	CYC TCPs	CIN TCPs
*Arabidopsis thaliana*	13	3	8
*Populus trichocarpa*	21	5	10
*Populus euphratica*	18	6	9
