# Supplementary material for: Genome-wide Identification of TCP Family Transcription Factors from Populus euphratica and Their Involvement in Leaf Shape Regulation
Source: Sci Rep. 2016 Sep 8;6:32795. doi: 10.1038/srep32795 (PMC5015053; doi:10.1038/srep32795)
Supplement: Supplementary Information [file srep32795-s1.pdf]

Genome-wide Identification of TCP Family Transcription Factors from *Populus euphratica* and Their Involvement in Leaf Shape Regulation

Xiaodong Ma[a,b,c], Jianchao Ma[d], Di Fan[e], Chaofeng Li [a,c], Yuanzhong Jiang[e] and Keming Luo[a, e*]

[a] Key Laboratory of Adaptation and Evolution of Plateau Biota, Northwest Institute of Plateau Biology, Chinese Academy of Sciences, Xining, 810008, China

[b] School of Chemistry and Chemical Engineering, Qinghai University for Nationalities, Xining, 810007, China

[c] University of the Chinese Academy of Sciences, Beijing, 100049 China

[d] State Key Laboratory of Grassland Agro-Ecosystem, School of Life Science, Lanzhou University, Lanzhou, China

[e] School of Life Science, Southwest University, Chongqing, 400715 China

* Corresponding author: Keming Luo, kemingl@swu.edu.cn.

Supplementary Table S1 TCP genes identified in *Populus trichocarpa* genome.

Name	Gene ID	Chromosome location	Exon/Intron number	Size/aa	Molecular weight	Isoelectric point
**PtrTCP1**	Potri.001G060000.1	Chr01:4606886..4608711 forward	1/0	302	32824.1	9.12
**PtrTCP2**	Potri.001G111800.1	Chr01:8915776..8918193 forward	1/0	397	41566.69	5.92
**PtrTCP3**	Potri.001G327100.1	Chr01:33128474..33130288 reverse	1/0	320	33881.91	8.99
**PtrTCP4**	Potri.001G375800.1	Chr01:39070007..39073153 reverse	1/0	413	45260.12	6.27
**PtrTCP5**	Potri.002G152200.1	Chr02:11505495..11507288 reverse	1/0	348	37322.96	9.51
**PtrTCP6**	Potri.003G120200.1	Chr03:14262872..14264067 reverse	4/3	287	30513.49	6.52
**PtrTCP7**	Potri.003G167900.1	Chr03:17835407..17836527 reverse	1/0	302	32569.92	6.23
**PtrTCP8**	Potri.004G046300.1	Chr04:3514571..3516334 forward	1/0	395	42703.64	7
**PtrTCP9**	Potri.004G065800.5	Chr04:5518337..5526108 forward	1/0	473	51719.14	7.88
**PtrTCP10**	Potri.004G116100.1	Chr04:10737586..10740883 forward	1/0	365	40352.07	7.19
**PtrTCP11**	Potri.004G222100.1	Chr04:22846150..22848314 forward	1/0	581	61311.54	6.74
**PtrTCP12**	Potri.005G090300.2	Chr05:6716502..6718585 reverse	1/0	272	27682.92	9.72
**PtrTCP13**	Potri.005G140600.1	Chr05:11788531..11788653 forward	1/0	41	4787.43	9.62
**PtrTCP14**	Potri.006G125800.1	Chr06:10151379..10152438 forward	1/0	198	21310.74	6.5
**PtrTCP15**	Potri.008G115800.1	Chr08:7411585..7413806 reverse	1/0	453	50789.88	6.58
**PtrTCP16**	Potri.009G009400.1	Chr09:1695921..1696474 forward	1/0	178	19484.71	5.59
**PtrTCP17**	Potri.010G130200.1	Chr10:14537655..14539110 forward	1/0	472	52976.54	8.94
**PtrTCP18**	Potri.011G055500.1	Chr11:4839143..4841262 forward	2/1	459	49920.59	7.42
**PtrTCP19**	Potri.011G083100.4	Chr11:8357434..8364561 reverse	1/0	478	51623.06	7.76
**PtrTCP20**	Potri.011G096600.2	Chr11:11763733..11766342 reverse	1/0	408	44468.04	6.49
**PtrTCP21**	Potri.012G059900.1	Chr12:6319154..6321338 reverse	2/1	394	44959.21	8.6
**PtrTCP22**	Potri.012G135900.1	Chr12:15188758..15189871 forward	1/0	357	38078.04	6.9
**PtrTCP23**	Potri.013G110700.1	Chr13:12370950..12373420 reverse	1/0	415	44095.33	7.2
**PtrTCP24**	Potri.013G119400.1	Chr13:13248572..13251015 forward	1/0	345	38049.44	5.94
**PtrTCP25**	Potri.014G078500.1	Chr14:6307930..6309239 forward	2/1	396	42892.04	9.58
**PtrTCP26**	Potri.015G050500.1	Chr15:5563400..5565125 forward	2/1	402	46041.59	6.35
**PtrTCP27**	Potri.015G058800.1	Chr15:8042378..8044079 forward	1/0	377	41264.43	7.77
**PtrTCP28**	Potri.015G138200.1	Chr15:14686905..14687993 forward	2/1	327	35283.41	6.09
**PtrTCP29**	Potri.016G074200.1	Chr16:5609370..5611923 forward	5/4	445	47611.13	5.97
**PtrTCP30**	Potri.016G094800.1	Chr16:8490852..8492588 reverse	1/0	200	21540.81	9.38
**PtrTCP31**	Potri.017G094800.1	Chr17:11175983..11178060 forward	2/1	333	36837.37	7.19
**PtrTCP32**	Potri.017G112000.1	Chr17:12758702..12760734 reverse	2/1	374	42177.97	9.31
**PtrTCP33**	Potri.019G081800.1	Chr19:11505280..11507506 reverse	1/0	417	44556.66	6.52
**PtrTCP34**	Potri.019G091300.1	Chr19:12229257..12232192 forward	1/0	346	38045.36	6.26
**PtrTCP35**	Potri.T044100.1	scaffold_41:210714..212429 forward	1/0	321	34146.02	8.99
**PtrTCP36**	Potri.T146100.1	scaffold_457:7249..10019 reverse	1/0	364	40352.07	7.19

Supplementary Table S2 TCP genes identified in *Populus euphratica* genome

Name	Gene ID	Exon/Intron number	Size/aa	Molecular weight	Isoelectric point
**PeuTCP1**	CCG000586.1	2/1	426	48070.51	6.44
**PeuTCP2**	CCG001135.1	2/1	374	42259.97	9.32
**PeuTCP3**	CCG002481.1	1/0	476	52186.43	7.38
**PeuTCP4**	CCG002729.1	2/1	405	45962.78	8.24
**PeuTCP5**	CCG003334.1	1/0	271	27639.92	9.72
**PeuTCP6**	CCG005156.1	1/0	200	21374.82	9.38
**PeuTCP7**	CCG006044.1	1/0	360	38316.84	6.05
**PeuTCP8**	CCG006216.1	5/4	623	68064.55	7.07
**PeuTCP9**	CCG007195.1	1/0	312	33041.71	7.17
**PeuTCP10**	CCG007701.1	1/0	363	40361.95	7.74
**PeuTCP11**	CCG007964.1	1/0	414	45293.21	6.46
**PeuTCP12**	CCG009717.1	1/0	320	33893.85	8.99
**PeuTCP13**	CCG010631.1	1/0	332	35489.13	9.96
**PeuTCP14**	CCG011685.1	1/0	478	51802.22	8.19
**PeuTCP15**	CCG012390.1	1/0	377	41285.37	6.39
**PeuTCP16**	CCG012899.1	1/0	346	38129.38	6.23
**PeuTCP17**	CCG013012.1	5/4	444	47806.3	5.84
**PeuTCP18**	CCG015462.1	3/2	350	38835.65	9.55
**PeuTCP19**	CCG018013.1	1/0	274	27955.01	9.51
**PeuTCP20**	CCG018118.1	1/0	176	19262.68	6.31
**PeuTCP21**	CCG019123.2	2/1	299	32062.04	9.15
**PeuTCP22**	CCG020734.1	1/0	278	30140.78	9.14
**PeuTCP23**	CCG021224.1	1/0	478	51819.21	8.19
**PeuTCP24**	CCG023727.1	1/0	199	21441.89	6.3
**PeuTCP25**	CCG024429.1	1/0	396	41441.54	5.71
**PeuTCP26**	CCG025130.1	1/0	472	52937.45	8.8
**PeuTCP27**	CCG026334.1	1/0	364	40420.12	7.21
**PeuTCP28**	CCG026357.1	1/0	414	43815.27	7.87
**PeuTCP29**	CCG026590.1	1/0	346	37988.42	6.09
**PeuTCP30**	CCG026656.1	1/0	417	44523.66	6.52
**PeuTCP31**	CCG028420.1	2/1	400	45920.55	7.08
**PeuTCP32**	CCG031555.1	1/0	278	29918.78	6.53
**PeuTCP33**	CCG032104.1	1/0	395	42646.71	6.9

Supplementary Table S3 Primers used in qRT-PCR

Primer Name	Sequence (5’-3’)
P. trichocarpa	
**PtrTCP1-qF**	CCTGCCTCTGAGGACAAGAAA
**PtrTCP1-qR**	TGACACAACACTCCCCCAAT
**PtrTCP2-qF**	CCACGCTGATCAAAATCGTAGG
**PtrTCP2-qR**	ACCACACTACTGTTCATGGCT
**PtrTCP3-qF**	TCAAAGAGGAGTAGAGGACCCA
**PtrTCP3-qR**	GCCCTTGGGATCCATGAACA
**PtrTCP4-qF**	CAGCACCACACACCAGAAAT
**PtrTCP4-qR**	TCTGTAGCGGTGGTAGTGGT
**PtrTCP5-qF**	TTGTTAAGTGGGGCCTGCAT
**PtrTCP5-qR**	CATTAGCAGGGACCACCAGT
**PtrTCP6-qF**	ATTGCTTTGCTGCTGCCGAC
**PtrTCP6-qR**	CAAATATTGGAGCTGCGGTGG
**PtrTCP7-qF**	ACAGTAAAGCGTACATTCCCA
**PtrTCP7-qR**	GAAAGGCAGAAAAGCACCTGT
**PtrTCP8-qF**	TAAAGCTCGTCCAGCTCTTCC
**PtrTCP8-qR**	ATAGGATACTGTGTCGAGGGG
**PtrTCP9-qF**	AGCCTTGCTGCTGGTTTCAA
**PtrTCP9-qR**	CGTTTGTGGTGGATCCGTCTA
**PtrTCP10-qF**	TCACATGCCACAGCTTCCAT
**PtrTCP10-qR**	GTCCGGTGGACTTTCGCTAT
**PtrTCP11-qF**	AACTCAAGAGCCCCACCAAT
**PtrTCP11-qR**	AGCCCATGAAAGGACCTTGA
**PtrTCP12-qF**	TTGTTTTTCGGGGTGCCTGT
**PtrTCP12-qR**	CGCATAAGGCAATGGCACA
**PtrTCP13-qF**	TACGACAACAACAACAAGACGC
**PtrTCP13-qR**	AAATTGGAAGATCCCGGCAG
**PtrTCP14-qF**	CCGTGTTACAGTGCAGGGTC
**PtrTCP14-qR**	AGAAGCCACGAGGATTGAGG
**PtrTCP15-qF**	TGCTCGGCTTCGATAAAGCA
**PtrTCP15-qR**	CAAACACTCTTGCCGTCAGC
**PtrTCP16-qF**	GAGATCGTCGTATCCGCCTG
**PtrTCP16-qR**	AGCCACTCAATGGTCTCACC
**PtrTCP17-qF**	GCGAACAGGGAAGAAGGACA
**PtrTCP17-qR**	TGCTTTATCGAAGCCGAGCA
**PtrTCP18-qF**	GATACAACAGCACCCAAAAGAGA
**PtrTCP18-qR**	TGTGGGAATTTCTCCAATGGC
**PtrTCP19-qF**	TGGTGGAAAAGATCGGCACA
**PtrTCP19-qR**	CAACCGATCTTGGAGGTCGT
**PtrTCP20-qF**	GTGGAGGAAGTGCAGGACTC

**PtrTCP20-qR**	CGGGTCTATCCAAGCACGAA
**PtrTCP21-qF**	CGTACTCCAGAGGGCATGAT
**PtrTCP21-qR**	TCTGAGAATTGATGCTCCTGAT
**PtrTCP22-qF**	CGAGTGCTAGTACTGGTGGC
**PtrTCP22-qR**	ACGCAAGCCAGTCATCTCAA
**PtrTCP23-qF**	CCTGTGCTAATGCCTGTCGAT
**PtrTCP23-qR**	GAACTAGTGCAGACATGGGACA
**PtrTCP24-qF**	GAGCTCTTTCGGCTCAACTCT
**PtrTCP24-qR**	ATTGTGGGTTTGCAGCAATAG
**PtrTCP25-qF**	GGTGATTGGCAGGTACAAACC
**PtrTCP25-qR**	TCATGCTCGCATCTTCTCCA
**PtrTCP26-qF**	TGAAGCTGAAGTGCCGTTCT
**PtrTCP26-qR**	TGAATTGATTCTCTTGAGGAATTCCA
**PtrTCP27-qF**	GATGGGCAGGTGCAAAACAG
**PtrTCP27-qR**	CGGGGACCTGTCCAGTAGTA
**PtrTCP28-qF**	AGGGAGTAGTGTCGGTGTTG
**PtrTCP28-qR**	AGTACTTGATCCGGCCAAT
**PtrTCP29-qF**	ATGTGCCTGGTCCTGAAG
**PtrTCP29-qR**	GTGCACTTTCTGATGCCGTT
**PtrTCP30-qF**	GTTCGCTACCAACCCTGCT
**PtrTCP30-qR**	AGTCTCGCTCCAGCTAATCC
**PtrTCP31-qF**	CACCTGGTACAACACAGTCT
**PtrTCP31-qR**	AACTGTATGGTATGGACTCG
**PtrTCP32-qF**	CATCTGCAACAATGGGTTATCA
**PtrTCP32-qR**	AGTTGTATTTCTGTAGACGGA
**PtrTCP34-qF**	AAAGGGCAGTGTGTTTGGGG
**PtrTCP34-qR**	TACCCACCACTCCTACCCAC
**PtrTCP35-qF**	TGTCTGCTACACGTGCTGAT
**PtrTCP35-qR**	GCAGACATAAACGGCTCTTTGG
**PtrTCP36-qF**	GTGCACAGAGGAAGTAGAGGA
**PtrTCP36-qR**	ATCCGGTGTCAAAACTGCCT
	P. euphratica
**PeuTCP3F**	AGATCTATGGAGGCGGAGGAGATTCAG
**PeuTCP3R**	GCTAGCTCAGTTCTTCCCTTTGCCTTTC
**PeuTCP4F**	AGATCTATGTTTCCATTAAGCTACAATGCC
**PeuTCP4R**	ACTAGTTCAAGGGTGGTCAGAGAGATG
**PeuTCP5F**	AGATCTATGTCAGATAACAGTGGTGCTGT
**PeuTCP5R**	ACTAGTTCAACGCTGATCCTCCTCTC
**PeuTCP6F**	AGATCTATGGCTTCTGAAACGGTTCTCTA
**PeuTCP6R**	ACTAGTCTACATCTTATGCTCCCCAGTTA
**PeuTCP8F**	AGATCTATGGAAGGAGGTGATGATCAGC
**PeuTCP8R**	ACTAGTTTAAAAACTAATGGCATAATTCGA
**PeuTCP11F**	AGATCTATGGGAGAGAGCCACCACCA
**PeuTCP11R**	ACTAGTTCAATGGCGAGAATCAGAGGAAG

**PeuTCP12F**	AGATCTATGGATCCCAAGGGCTCTAAC
**PeuTCP12R**	ACTAGTTCACTGCCCTGATCCTTGTG
**PeuTCP21F**	AGATCTATGGCATCGACACAAAAGCAG
**PeuTCP21R**	ACTAGTTCAAGATCGAGACATGAACTGT
**PeuTCP27F**	AGATCTATGATTACTAGTTCAAAGCAAGTGG
**PeuTCP27R**	TCTAGATCATTTACAAGGGAATTGTGGGT
**PeuTCP29F**	AGATCTATGAAGAACACCGGAGGAGA
**PeuTCP29R**	ACTAGTTCAATTCTGAGAATTTGGAGAAGAGG
**PeuTCP33F**	AGATCTATGGAAGGAGGTGATGATCAGT
**PeuTCP33R**	ACTAGTTTATGAGTGATGACTCGTTGT
**PeuSPL8F**	AGCAGCTCTCTCCCTATGGT
**PeuSPL8R**	GGGTTTGCCTCTCTTGCCTA
**PeuSPL6F**	CGTCAATGGACTGCAGCAAC
**PeuSPL6R**	TGTGCCCTGCTAAACGTCTC
**PeuSPL17F**	CGCCTAGCTGGCCATAATGA
**PeuSPL17R**	CTCAGAAGACCTTGGAGCGG
**PeuSPL23F**	TGGTGGTAGTGGTAGGTCGT
**PeuSPL23R**	ACTCAGATAAATCATGGAACCTGC
**PeuSPL27F**	CACTAGTGTGGTGGAGAGCG
**PeuSPL27R**	GGACCCGTCACCTGCATAAA

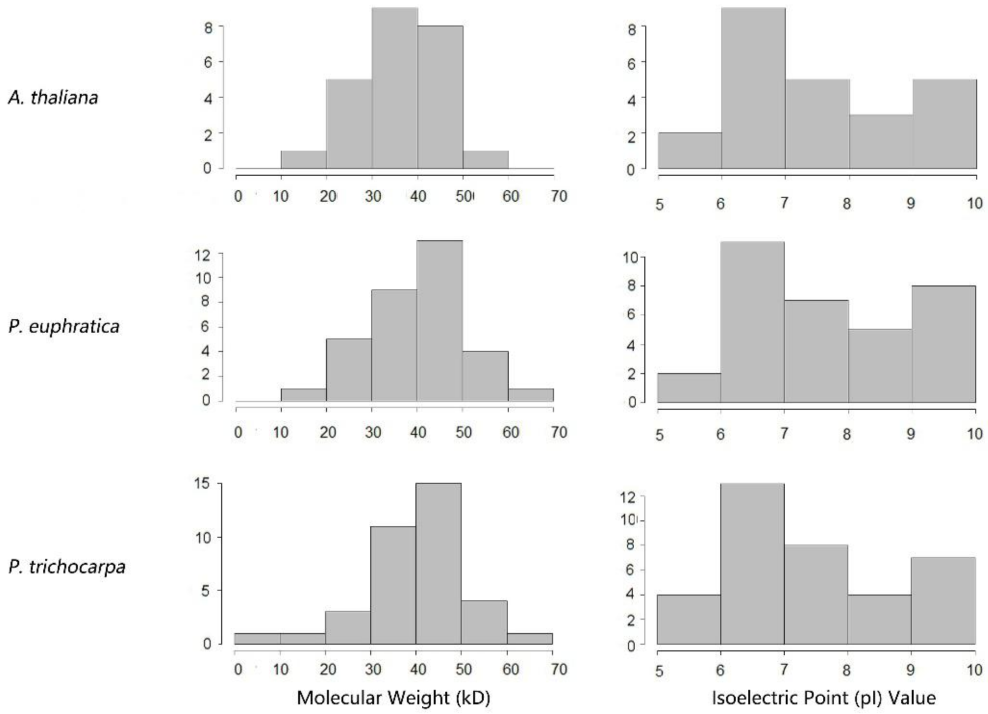

Supplementary Figure S1. Distribution of Molecular weight and isoelectric point of TCP proteins from *Arabidopsis*, *P. euphratica* and *Populus trichocarpa*.

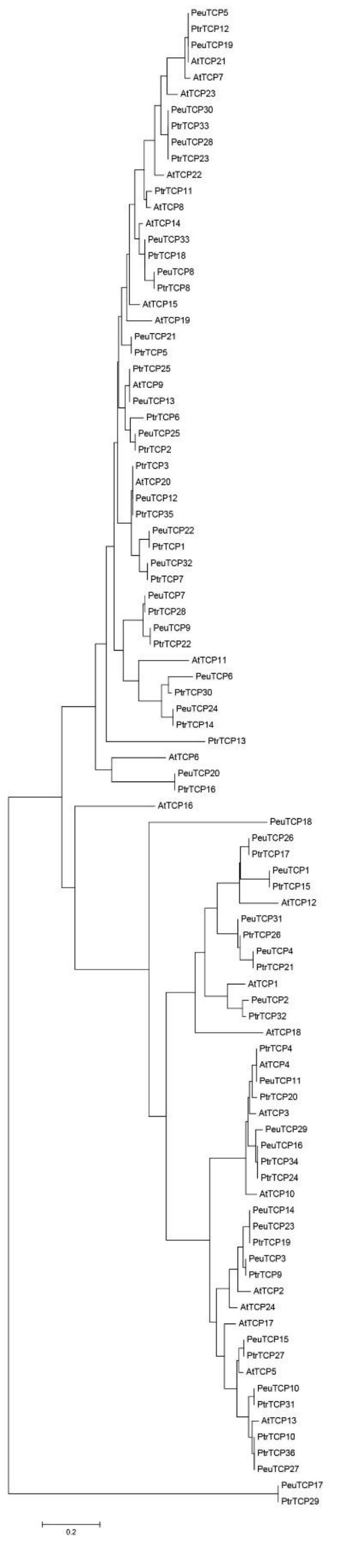

Supplementary Figure S2. Phylogenetic tree of TCPs built with the minimal evolution method

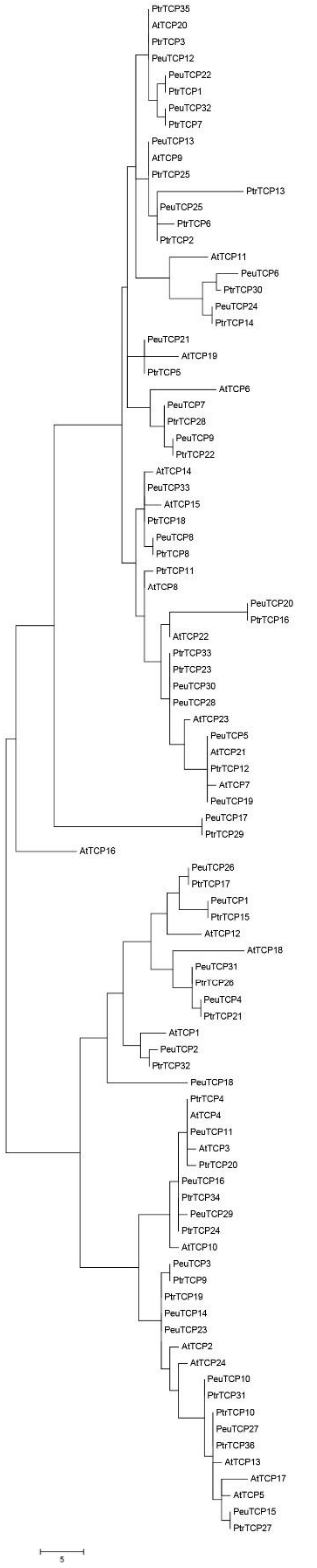

Supplementary Figure S3. Phylogenetic tree of TCPs built with the maximal parsimony method

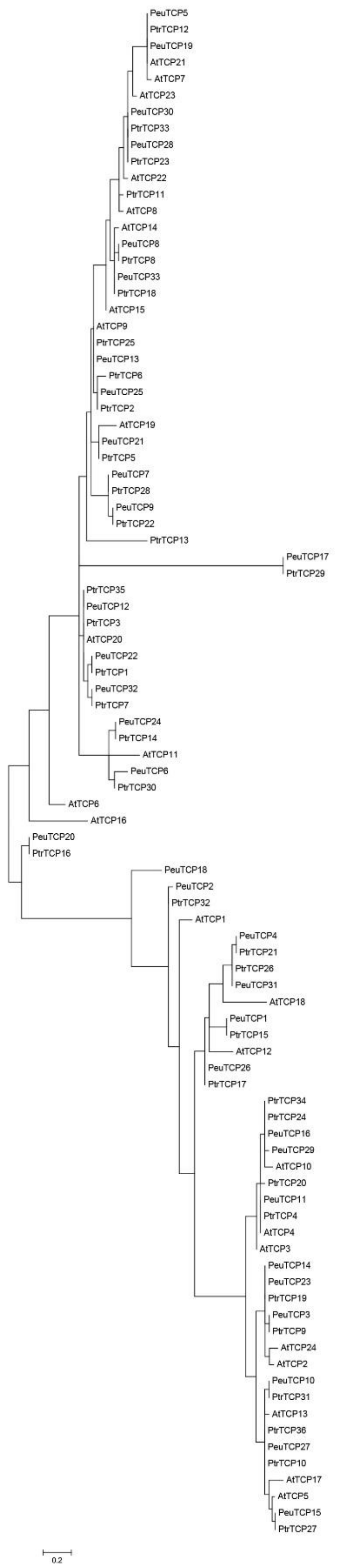

Supplementary Figure S4. Phylogenetic tree of TCPs built with the neighbour-joining method

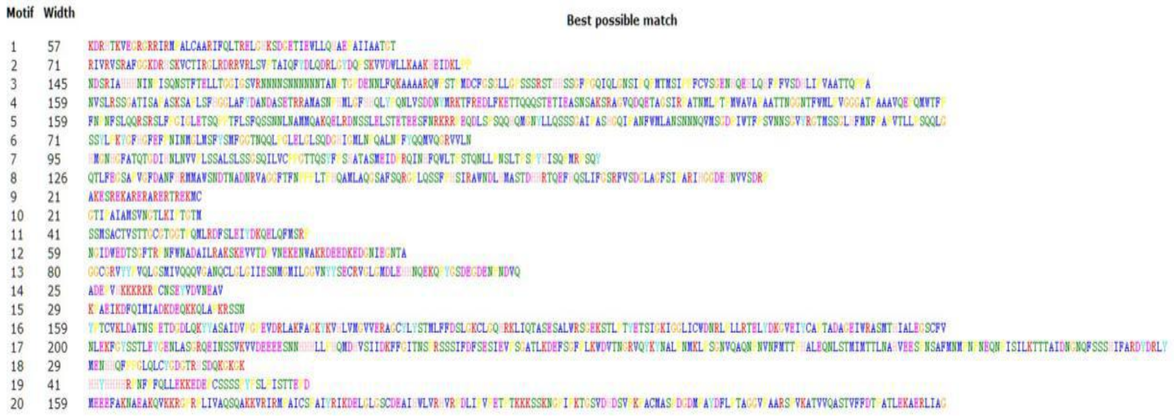

Supplementary Figure S5. Protein motifs identified by MEME

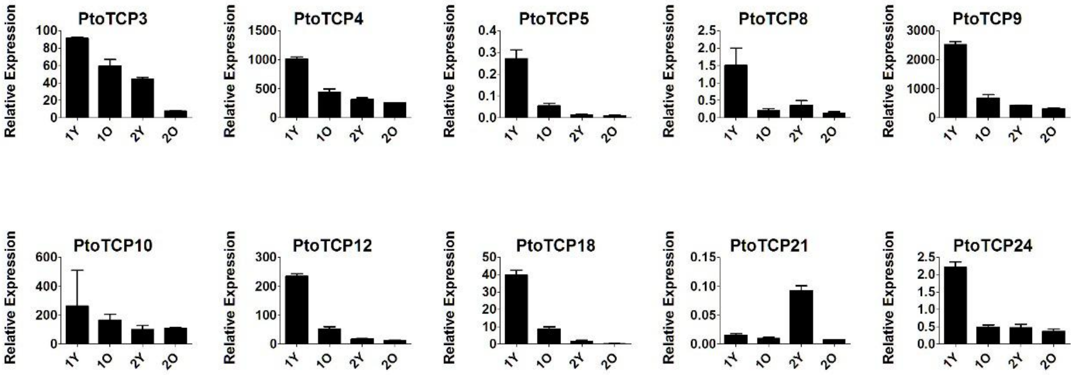

Supplementary Figure S6. Expression pattern of selected TCP genes in *P. tomentosa* leaves. Each bar represents three biological replicates and error bar represents S.D.

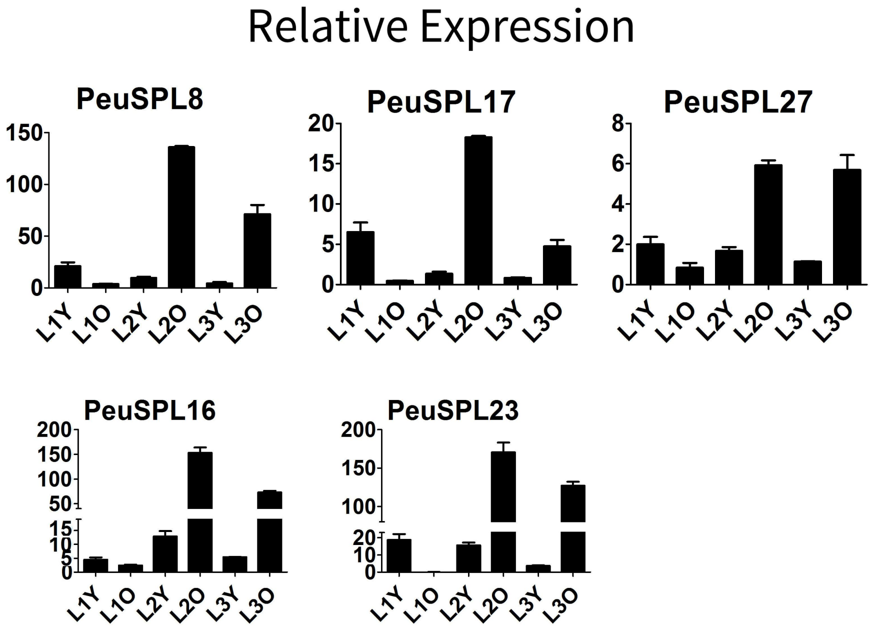

Supplementary Figure S7. Expression pattern of selected SPL genes in *P. euphratica*. Each bar represents three biological replicates and error bar represents S.D.

Reference

1 Li, C. & Lu, S. Molecular characterization of the SPL gene family in *Populus trichocarpa*. *BMC Plant Biology* **14**, 1 (2014).
